# Swertianin Suppresses M1 Macrophage Polarization and Inflammation in Metabolic Dysfunction-Associated Fatty Liver Disease via PPARG Activation

**DOI:** 10.3390/genes16060693

**Published:** 2025-06-06

**Authors:** Jing Xia, Wei Xiong, Ce Yang, Ying Tan, Xiaoyuan Peng, Wenxiang Wang

**Affiliations:** 1 Chongqing Key Laboratory of Development and Utilization of Genuine Medicinal Materials in Three Gorges Reservoir Area, Chongqing 404120, China; xiajing0510@163.com (J.X.); xiongweichn202202@163.com (W.X.); yangce2024@163.com (C.Y.); daisy_tanying@163.com (Y.T.); pengxiaoyuan2023@163.com (X.P.); 2 Department of Basic Medical Sciences, Chongqing Three Gorges Medical College, Chongqing 404120, China; 3 College of Pharmacy, Chongqing Three Gorges Medical College, Chongqing 404120, China; 4 College of Clinical Medicine, Chongqing Three Gorges Medical College, Chongqing 404120, China

**Keywords:** metabolic dysfunction-associated fatty liver disease, Swertianin, macrophage polarization, peroxisome proliferator-activated receptor-gamma, inflammation

## Abstract

**Background:** Metabolic dysfunction-associated fatty liver disease (MASLD) is closely associated with immune dysregulation and macrophage-driven inflammation. The activation of PPARG plays a critical role in modulating macrophage polarization and lipid metabolism, suggesting its potential as a therapeutic target for MASLD. **Methods:** We used UPLC-Q/TOF-MS and network pharmacology to investigate the key components and targets of Swertia davidi Franch, focusing on Swertianin. In vitro experiments on macrophages were conducted to assess the modulation of M1 polarization, and a mouse model of MASLD was utilized to explore the therapeutic effects of Swertianin. **Results:** Swertianin activated PPARG, leading to significant inhibition of M1 macrophage polarization, a reduction in lipid accumulation, and decreased inflammatory marker levels both in vitro and in vivo. The treatment significantly improved liver pathology in mice, indicating its therapeutic potential for MASLD. **Conclusions:** Swertianin’s activation of PPARG provides a novel mechanism for treating MASLD, targeting both macrophage polarization and inflammation.

## 1. Introduction

With the rising prevalence of obesity and type 2 diabetes, metabolic dysfunction-associated steatotic liver disease (MASLD) has become one of the most common chronic liver diseases, with a continuously increasing incidence that warrants serious attention [1,2,3]. MASLD encompasses a full spectrum of liver conditions, from simple steatosis to non-alcoholic steatohepatitis (NASH), fibrosis, cirrhosis, and hepatocellular carcinoma. In MASLD, hepatic fat accumulation can trigger inflammatory responses that progress to fibrosis and eventually cirrhosis if left uncontrolled [1]. Beyond liver-specific complications, MASLD is also associated with a significantly increased risk of cardiovascular events, such as coronary artery disease, atherosclerotic cardiovascular disease, and heart failure [4]. The pathophysiological mechanisms of MASLD are complex and involve genetic predisposition, metabolic dysregulation, oxidative stress, and cytokine signaling [5,6]. M1-type macrophages play a crucial role in the inflammatory processes and liver injury associated with MASLD by releasing pro-inflammatory cytokines such as TNF-α, IL-6, and IL-1β, which exacerbate hepatic inflammation and fibrosis [7,8].

Due to its complex pathophysiological mechanisms, the treatment options for MASLD remain highly limited [9,10,11]. The preferred treatment strategy is to control weight and blood sugar through lifestyle modifications, such as dietary adjustments and increased physical activity [3,12,13]. However, the effectiveness of these methods largely depends on the patient’s long-term self-management and discipline, and their effectiveness is limited in the advanced stages of the disease, varying from person to person [10,14]. In terms of pharmacological treatments, there are currently no FDA-approved drugs specifically for treating MASLD. The complex pathophysiological mechanisms of MASLD have led many trials to fail in meeting primary research endpoints, and clinically, drugs for managing related metabolic diseases, such as statins and insulin sensitizers, are commonly used [9,15,16]. Therefore, exploring new therapeutic targets and strategies is urgently needed.

In Chinese medicine, a variety of herbs and formulae have been used to regulate immune and inflammatory responses, showing potential therapeutic effects on chronic liver diseases [17]. Swertia davidi Franch, a traditional Chinese medicine, has become a research focus because of its unique chemical composition and potential pharmacological activities [18]. Swertianin, as one of the main active components of Swertia davidi Franch, is drawing scientific attention because of its anti-inflammatory and antioxidant properties.

The PPARG pathway is an important member of the nuclear receptor superfamily and is widely involved in pathological processes such as lipid metabolism and inflammation [19]. PPARG is activated by binding with ligands, subsequently forming a heterodimer with the retinoid X receptor, binding to peroxisome proliferator response elements on DNA, thereby regulating the transcription of target genes [20]. In MASLD and NASH diseases, the activation of PPARG shows potential therapeutic effects by regulating lipid metabolism and reducing inflammation [21]. Based on the important function of PPARG, various reversible covalent regulators targeting PPARG have been developed, showing high efficacy and safety in treating type 2 diabetes and reversing liver steatosis [22]. PPARG can influence macrophage polarization by regulating their metabolic state. For example, activation of PPARG promotes fatty acid oxidation in macrophages, supporting M2 polarization [23].

The main purpose of this study is to explore the role of Swertianin in MASLD and its mechanisms, particularly its potential to inhibit M1-type macrophage polarization by activating the peroxisome proliferator-activated receptor-γ (PPARG) pathway. By integrating various methods such as UPLC-Q/TOF-MS, network pharmacology, biochemical analysis, and animal models, this study aims to comprehensively assess the effects of Swertianin on improving hepatic lipid deposition and inflammatory responses.

## 2. Materials and Methods

### 2.1. Swertia Davidi Franch Acquisition

Swertia davidi Franch, belonging to the Gentianaceae family and the Swertia genus, is a perennial herb. It is primarily distributed in eastern Sichuan, western Hubei, western Hunan, and Jingdong of Yunnan in China. It is cultivated by manually planting wild seedlings with a height of 1.0–2.0 cm, collected daily, in sandy soil with a substrate moisture content of 40–50%. Swertia davidi Franch was extracted using 50% methanol. After ultrasonic extraction for 30 min, the sample was filtered through a 0.22 μm membrane and directly injected into the UPLC system for analysis.

### 2.2. Chemical Composition Analysis of Swertia Davidi Franch Using UPLC-Q/TOF-MS

This study conducted a detailed analysis of the chemical composition of Swertia Davidi Franch using UPLC-Q/TOF-MS. Initially, sample analysis was carried out using an Acquity UPLC H-Class ultra-high-performance liquid chromatography system (Waters, Milford, MA, USA) coupled with Xevo G2-S time-of-flight mass spectrometer (Waters, Milford, MA, USA). The chromatographic separation employed a BEH C18 column (2.1 × 50 mm, 1.7 μm, Waters, USA) with acetonitrile (ACN, Thermo Fisher Scientific, Waltham, MA, USA) and water containing 0.1% formic acid (HCOOH, Thermo Fisher Scientific, Waltham, MA, USA) as the mobile phase. The column temperature was set at 45 °C, the flow rate at 0.40 mL/min, and the injection volume controlled at 0.5 μL. For mass spectrometry analysis, electrospray ionization was used with scanning in both positive and negative ion modes. The capillary voltage was set at 2.5 kv, cone voltage at 25 V, ion source temperature at 120 °C, desolvation temperature at 400 °C, desolvation gas flow rate at 1000 L/hr, and MSe scan mode was employed. The stringent experimental conditions and parameter settings ensured the efficiency and accuracy of the chemical composition analysis of Swertia Davidi Franch [24]. Peaks were selected based on a signal-to-noise ratio (S/N) ≥ 10 to ensure high-confidence detection, and results were manually verified. Compound identification was performed using MS/MS fragmentation information and processed with UNIFI v.1.8 software. A total of 64 peaks were detected, among which 55 compounds were successfully matched with reference spectra and literature data, while 9 peaks remained unidentified. All UPLC-Q/TOF-MS analyses were conducted in three technical replicates.

### 2.3. Bioinformatics Analysis of Traditional Chinese Medicine

Following the completion of the UPLC-Q/TOF-MS chemical composition analysis, a bioinformatics analysis was conducted to identify potential biological targets of compounds in Swertia Davidi Franch. Representative compound data obtained from UPLC-Q/TOF-MS analysis of Swertia Davidi Franch were uploaded to the TCSMP database (https://old.tcmsp-e.com/tcmsp.php, accessed on 1 July 2025), and its built-in prediction tools were utilized to predict potential target interactions of the compounds. Based on the prediction results, important biological targets associated with the pathogenesis of MASLD were identified [25].

### 2.4. Public Data Download

In the initial phase of this study, to gain a comprehensive understanding of the molecular mechanisms of MASLD, the transcriptomic dataset GSE149863, related to MASLD, was downloaded from the Gene Expression Omnibus (GEO) database (https://www.ncbi.nlm.nih.gov/gds, accessed on 5 July 2025) using “Metabolic dysfunction-associated fatty liver disease” as the key search term. The dataset comprised 120 liver tissue samples from mice, with 60 normal samples and 60 MASLD samples. Simplifying this process, the GEOquery package in R language facilitated the direct downloading of the required dataset from the GEO database. Additionally, using “Metabolic dysfunction-associated fatty liver disease” as the key search term, disease targets related to MASLD were obtained from the GeneCards database (https://www.genecards.org/, accessed on 5 July 2025) [26].

### 2.5. Analysis of DEGs and GO/KEGG Pathway Enrichment Analysis

To identify key genes associated with MASLD, differential expression analysis was performed on the downloaded transcriptomic dataset. The analysis was conducted using the limma package in R (version 3.44.3). To maximize the identification of potential MASLD-related regulatory factors, the threshold for statistical significance was set at a *p*-value < 0.05 and |log_2_ fold change| > 0.5 to screen for significantly differentially expressed genes (DEGs) [27].

The screened DEGs were subjected to Gene Ontology (GO) and Kyoto Encyclopedia of Genes and Genomes (KEGG) pathway enrichment analysis to investigate their biological functions and involvement in metabolic pathways. Enrichment analysis was conducted using the clusterProfiler package (version 3.16.1). The Benjamini–Hochberg (BH) method was applied to adjust for the false discovery rate (FDR), reducing the risk of false positives due to multiple testing. Pathways with an adjusted *p*-value (FDR) < 0.05 were considered significantly enriched, and the most up-to-date databases were used for annotation.

### 2.6. Validation of Molecular Docking with AutoDock

To evaluate the binding affinity between the key active component Swertianin and core targets, molecular docking simulations were performed using AutoDock 4.2.6 (The Scripps Research Institute, San Diego, CA, USA). The 2D and 3D structures of Swertianin and the reference compound Rosiglitazone (PubChem CID: 5281661 and 77999, respectively) were retrieved from the PubChem database (https://pubchem.ncbi.nlm.nih.gov/, accessed on 5 July 2025) and energy-minimized using the MMFF94 force field in Chem3D. The crystal structure of PPARG was obtained from the RCSB Protein Data Bank (PDB ID: 3CS8), representing the ligand-binding domain of PPARG at a resolution of 2.30 Å.

Protein preprocessing included the removal of water molecules and native ligands, the addition of polar hydrogens, and the assignment of Gasteiger charges. During docking, the protein structure was kept rigid, and only the ligands were set as flexible. A rigid docking approach was employed. Based on previous studies, the docking grid was centered on the known ligand-binding site of PPARG, including key residues such as Asp396, Tyr320, and Arg397. No flexible side chains were defined. The docking was conducted using the Lamarckian Genetic Algorithm, with 50 runs per ligand. The conformation with the lowest binding energy was selected as the optimal binding pose.

### 2.7. Macrophage Culture and Treatment

To investigate the effects of Swertianin on macrophages, macrophages were initially cultured in the laboratory. Human monocytic cell line THP-1 cells (TIB-202, ATCC, Manassas, VA, USA) were used as precursors for macrophages and cultured in RPMI 1640 medium (C11875500BT, Gibco, Grand Island, NY, USA) supplemented with 10% fetal bovine serum (26140079, Thermo Fisher Scientific, Waltham, MA, USA) and 1% penicillin-streptomycin (100 U/mL penicillin and 100 μg/mL streptomycin, 15140122, Thermo Fisher Scientific, Waltham, MA, USA). THP-1 cells used in this study were between passages 3 to 10 and tested negative for mycoplasma contamination via PCR.

To induce differentiation into macrophages, 100 ng/mL of PMA (HY-18739, Phorbol 12-myristate 13-acetate, MedChemExpress, Monmouth Junction, NJ, USA) was added and incubated for 48 h. After differentiation, the macrophages were divided into two groups: a control group treated with 0.1% DMSO and a treatment group exposed to Swertianin (purity > 98%, self-extracted and validated by HPLC). Swertianin was dissolved in DMSO to prepare a 10 mM stock solution and diluted with culture medium to a final concentration of 10 μM. The cells were treated for 24 h [28,29].

### 2.8. CCK-8 Cytotoxicity Assay

THP-1 cells were seeded in 96-well plates at a density of 10^4^ cells/mL, treated with different concentrations of PMA (0 ng/mL–400 ng/mL) and Swertianin (0–35 μM), and cell viability was assessed after 24 h using the CCK-8 kit.

### 2.9. siRNA Transfection

SiRNAs were obtained from genepharma (Shanghai, China). The target sequences are listed below: si-NC: UUCUCCGAACGUGUCACGUTT, si-PPARG: GCCCTGGCAAAGCATTTGTAT. Lipofectamine RNAiMAX Transfection Reagent (Invitrogen, Thermo Fisher Scientific, Waltham, MA, USA) was used for transfection according to the manufacturer’s protocols.

### 2.10. Western Blot Analysis

To assess the impact of Swertianin on M1-type macrophage marker expression, a Western blot analysis was conducted. Cells from each group/mouse liver tissues were collected and lysed on ice for 30 min with RIPA lysis buffer containing 1% PMSF (P0013B, Beyotime, Shanghai, China), containing 1% PMSF. The buffer consisted of 50 mM Tris (pH 7.4), 150 mM NaCl, 1% Triton X-100, 1% sodium deoxycholate, 0.1% SDS, along with various inhibitors, including sodium orthovanadate, sodium fluoride, EDTA, and leupeptin, followed by centrifugation at 14,000× *g* at 4 °C to collect the supernatant. The protein concentration of the samples was determined using the BCA method (P0012S, Beyotime, Shanghai, China). A total of 50 μg of protein was denatured by boiling in a 5× loading buffer at 100 °C for 10 min. Electrophoresis was performed using a separating gel and a concentrating gel, and the protein bands of interest were transferred onto a PVDF membrane. The PVDF membrane was then blocked with 5% skim milk powder at room temperature for 1 h and subsequently incubated overnight at 4 °C with primary antibodies against iNOS (ab283655, Abcam, Cambridge, MA, USA), TNF-α (ab6671, Abcam, Cambridge, MA, USA), PPARG (ab209350, Abcam, Cambridge, MA, USA), and GAPDH (ab181602, Abcam, Cambridge, MA, USA), iNOS (ab15323, Abcam, Cambridge, MA, USA), TNF-α (ab183218, Abcam, Cambridge, MA, USA), PPARG (ab209350, Abcam, Cambridge, MA, USA), and GAPDH (ab181602, Abcam, Cambridge, MA, USA). GAPDH served as the loading control. After washing with phosphate-buffered saline with Tween (PBST) at room temperature, the membranes were incubated with Horseradish Peroxidase-conjugated goat anti-rabbit IgG secondary antibody (1:10,000, BA1056, Biodot, Wuhan, China) for 1 h at room temperature, followed by six washes with PBST for 5 min each. The membranes were then evenly treated with ECL reagent (AR1172, Biodot, Wuhan, China) and exposed to an imaging system (Amersham Imager 600, Marlborough, MA, USA) for chemiluminescence detection. Next, grayscale analysis was performed using ImageJ, v. 1.51, and GAPDH was used as an internal reference for normalization. The experiment was repeated three times.

### 2.11. RT-qPCR Detection

Total RNA was extracted using TRIzol reagent (15596018CN, Thermo Fisher Scientific, Waltham, MA, USA) in the RT-qPCR experiment. Subsequently, cDNA was synthesized using the cDNA synthesis kit (RR047A, Takara Bio Inc., Osaka, Japan), followed by quantitative PCR analysis on a real-time fluorescence quantitative PCR machine (A28575, Applied Biosystems, Waltham, MA, USA) using SYBR Green PCR Master Mix (4309155, Thermo Fisher Scientific, Waltham, MA, USA). The amplification conditions included 30 s denaturation at 95 °C, followed by 40 denaturation cycles at 95 °C for 5 s, annealing at 95 °C for 5 s, and extension at 60 °C for 34 s. The 2^−ΔΔCt^ method was used for relative quantification, with normalization to GAPDH as the internal control. PCR Primer Design for Cell Experiments Using Primer-BLAST Tool, the primer sequences for cell experiments are listed in Table 1, and those for mouse liver tissue qPCR are provided in Table 2 [30,31,32].

### 2.12. Oil Red O Staining

An Oil Red O staining experiment was conducted to assess the accumulation of lipids in macrophages and mouse liver. THP-1 macrophages were fixed in a 4% paraformaldehyde solution (30525-89-4, Sigma-Aldrich, USA) for 12 min and washed in PBS (AM9624, Sigma-Aldrich, St. Louis, MO, USA) for 15 s. Subsequently, the cells were stained with filtered Oil Red O solution (C0157S, Beyotime, Shanghai, China) at room temperature for 5 min and dispersed in a 60% isopropanol solution (563935, Sigma-Aldrich, St. Louis, MO, USA) for 10 s. After a 15-s wash with PBS, the cells were stained with Sudan III solution for 3 min. Frozen sections, previously prepared and stored at −20 °C, were retrieved from the freezer and reheated at room temperature for 5–10 min. The dye solution was prepared by mixing the stock solution and diluent at a ratio of 5:2, then filtered slowly through filter paper for later use. The frozen sections were placed in the staining solution for 15 min, then rinsed in distilled water at 37 °C for 15 s, restained for 5 min, and washed for 45 s. The stained cells and slides were observed under an inverted microscope (DMI4000 B, Leica Microsystems, Wetzlar, Germany), and the quantification of lipid droplets was conducted using image analysis software (ImageJ, v. 1.51) [31].

### 2.13. ELISA Detection

To determine the impact of Swertianin treatment on the production of inflammatory factors by macrophages and evaluate the expression levels of inflammatory factors in serum, an enzyme-linked immunosorbent assay (ELISA) was utilized. Supernatants from Swertianin-treated and untreated macrophage cultures were collected. Monoclonal antibodies targeting Endocan were coated on a 96-well microplate according to the instructions of human-specific ELISA kits (ab178013, IL-6; ab181421, TNF-α, Abcam, Cambridge, MA, USA) and mouse-specific kits (ab100713, IL-6; ab208348, TNF-α, Abcam, Cambridge, MA, USA). The plate was then incubated overnight at 4 °C, followed by sealing at room temperature for 1 h and washing with phosphate-buffered saline (PBS). Subsequent steps were performed according to the kit’s instructions. The optical density (OD) value was measured at a wavelength of 450 nm using a microplate reader (A51119500C, Thermo Fisher Scientific, Waltham, MA, USA) [33].

### 2.14. Construction of Animal Models

To investigate the therapeutic effect of Swertianin on MASLD, an MASLD mouse model was first established. Male C57BL/6 mice, aged 8 weeks (40 in total, purchased from Hunan Sleek Jinda Experimental Animal Co., Ltd., Changsha, China), were selected and raised under specific-pathogen-free (SPF) conditions, with a 12-h light/dark cycle and free access to water and food, and acclimated for 1 week at 24–26 °C. No abnormal behavior or signs of discomfort were observed throughout the entire handling process. The animals’ daily activities, grooming behavior, and feeding were within the normal range. The animal experiments were approved by the Animal Ethics Committee of Chongqing Three Gorges Medical College, approval number SYYZ-A-2403-0006.

The mice were randomly divided into four groups, each consisting of 10 mice: (1) normal diet (Control), (2) normal diet + Swertianin (Control-S), (3) high-fat diet (Model), and (4) high-fat diet + Swertianin (Model-S). The MASLD model was induced by feeding the mice a high-fat diet for four consecutive weeks (High-fat feed composition: 80.4% base feed + 2% cholesterol + 10% lard + 0.5% sodium cholate + 0.1% methylthiouracil + 5% sucrose + 2% egg yolk powder) (Methylthiouracil inhibits thyroid hormone synthesis, slowing fat metabolism and further promoting fat accumulation), while the control group received a normal diet (96.9% base feed + 3% sucrose) [34].

The mice in the Swertianin treatment group received Swertianin (10 mg/kg, orally, daily) for four weeks after model establishment. On the day before the end of the experiment, all groups of mice fasted for 12–14 h with free access to water. The mice were accurately weighed and anesthetized with 0.4 mL/kg of 3% pentobarbital (P0500000, Sigma-Aldrich Corporation, St. Louis, MO, USA), and peripheral blood was collected. The blood samples were allowed to stand at room temperature for 90 min, then centrifuged at 4 °C, 2500 rpm for 20 min to collect serum, which was stored at −80 °C [35].

Following the opening of the abdominal cavity to observe liver morphology and texture, the liver wet weight was measured. Liver tissues from the same region were fixed in 4% paraformaldehyde and embedded in an O.C.T. compound. The remaining liver tissues were rapidly frozen in liquid nitrogen and stored at −80 °C for triglyceride (TG) and protein signaling pathways related to lipid metabolism analyses [36].

### 2.15. NAS Score

The NAS scoring system is a semi-quantitative method used to assess the severity of MASLD and NASH. Steatosis is scored from 0 to 3 based on the extent of hepatic lipid accumulation. The number of necrotic foci is counted under a 20× microscope, with a score ranging from 0 to 3. Hepatocellular ballooning is scored from 0 to 2 based on severity. The final NAS is the sum of the individual scores [37].

### 2.16. Biochemical Analysis

To assess the accumulation of fat in the livers of mice, biochemical analyses of TG and total cholesterol (TC) were performed. At the conclusion of the experiment, mouse serum was collected, and TG and TC levels were determined using the TG assay kit (ab65336, Abcam, USA) and the TC assay kit (BC1985, Solarbio, Seattle, WA, USA), respectively. The measurements were conducted on a fully automated biochemical analyzer (Beckman Coulter, Indianapolis, IN, USA) [36].

### 2.17. H&E Staining

Hematoxylin and eosin (H&E) staining was carried out to observe the pathological changes in liver tissue. Mouse liver tissue samples were fixed in 4% paraformaldehyde (30525-89-4, Sigma-Aldrich, St. Louis, MO, USA) for 30–50 min. Subsequently, the samples underwent dehydration, clearing, and paraffin embedding. The tissue sections were sliced to a thickness of 5 μm, stained with hematoxylin and eosin (15086-94-9, Sigma-Aldrich, St. Louis, MO, USA) for 5 min, differentiated with 1% hydrochloric acid, and then counterstained with eosin for 3 min. The sections were dehydrated, cleared, and coverslipped. The stained sections were observed under an optical microscope (DMI4000 B, Leica Microsystems, Germany) [36].

### 2.18. Immunofluorescence

Liver tissue sections were prepared and incubated with rabbit anti-PPARG primary antibody (ab209350, Abcam, USA), anti-F4/80 (14-4801-82, Thermo Fisher Scientific, Waltham, MA, USA), and anti-CX3CR1 (702321, Thermo Fisher Scientific, Waltham, MA, USA) at 4 °C for 12 h, followed by secondary antibody (1:200, Beyotime, China) for 1 h. DAPI staining was used for nuclear counterstaining, and the sections were observed under a Leica microscope (DMI4000 B, Leica Microsystems, Germany). Subsequently, ImageJ software was utilized to calculate the proportion of positive proteins [38].

### 2.19. Flow Cytometry to Assess M1 Polarization (CD86, iNOS, TNF-a)

Liver tissues from each group of mice were collected, cut into small pieces, and incubated in a dissociation medium containing 100 units/mL collagenase IV and 50 μg/mL DNase I at 37 °C for 30 min. The cell suspension was filtered through a 70 μm filter and washed with PBS. Red blood cells were lysed on ice for 10 min using RBC lysis buffer (420301, BioLegend, San Diego, CA, USA). Subsequently, the cells were incubated with the relevant antibodies at room temperature for 25 min. The mouse antibody APC anti-CD86 (1:100; 12-0862-82) was obtained from Thermo Fisher Scientific (USA), anti-iNOS (1:100, MA5-16422, Thermo Fisher Scientific, Waltham, MA, USA), anti-TNF-a (1:100, PA1-40281, Thermo Fisher Scientific, Waltham, MA, USA). NK cells were detected using a FACSAria II flow cytometer (BD Biosciences, San Jose, CA, USA), and these data were analyzed using FlowJo software (Tree Star) v11 [39,40,41].

### 2.20. Statistical Analysis

All Data were obtained from at least three independent experiments and presented as mean ± standard deviaion (SD). For comparisons between the two groups, an independent samples *t*-test was applied. When comparing three or more groups, one-way analysis of variance (ANOVA) was performed. If the overall ANOVA revealed significant differences, Tukey’s honest significant difference (HSD) test was used for post hoc multiple comparisons. In cases where data did not meet normality assumptions or showed unequal variances, non-parametric tests such as the Mann–Whitney U test or Kruskal–Wallis test were used instead. Statistical analyses were conducted using GraphPad Prism version 9 (GraphPad Software) and R programming language (version 3.44.3). A two-sided *p* value below 0.05 was considered statistically significant.

## 3. Results

### 3.1. Screening Key Chemical Components in Swertia Davidi Franch Using UPLC-Q/TOF-MS

This study utilized ultra-performance liquid chromatography-tandem quadrupole time-of-flight mass spectrometry (UPLC-Q-TOF-MS) to conduct a rapid and comprehensive qualitative analysis of the chemical constituents in the 50% methanol extract of Swertia davidi Franch. Data processing involved manual interpretation coupled with database matching in identifying compounds in Swertia davidi Franch based on accurate mass ions, secondary fragments, and other information. The preliminary identification results and the relevant literature on the Gentianaceae family were used to construct a database of potential compounds present in Swertia davidi Franch. This custom compound database was then imported into the UNIFI software for matching and validation, facilitating the structural identification of the main chemical components in Swertia davidi Franch.

**Figure 1:**
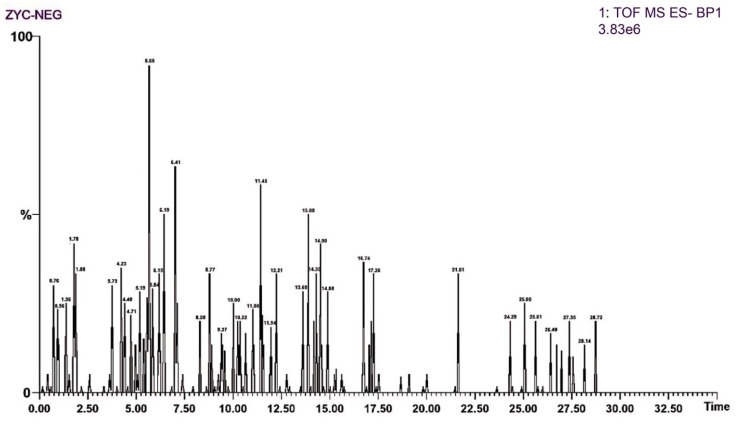
Negative ion mode mass spectrometry data of Swertia davidi Franch extract. Note: Displaying mass spectrometry data of Swertia davidi Franch extract in negative ion scan mode, highlighting its high response values and detection accuracy.

**Figure 2:**
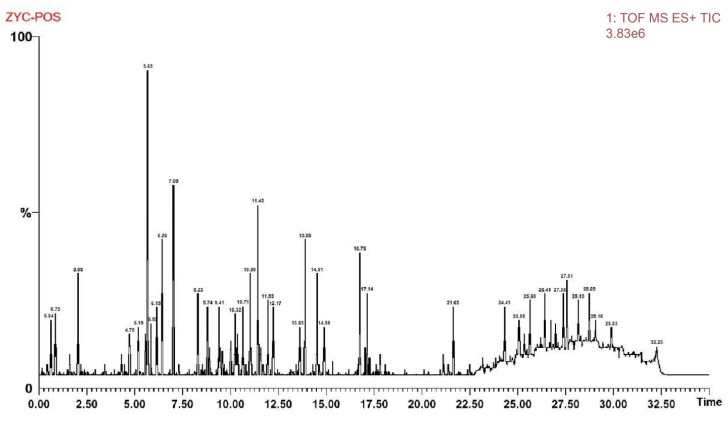
Positive ion mode mass spectrometry data of Swertia davidi Franch extract. Note: Showing mass spectrometry data of Swertia davidi Franch extract in positive ion scan mode, emphasizing its high response values and detection quality.

Regarding mass spectrometric data, the response values in negative ion scan mode were superior to those in positive ion mode, demonstrating good mass accuracy that fully met the requirements for identifying molecular formulas and fragment compositions based on precise mass numbers (Figure 1 and Figure 2). The majority of compounds exhibited better responses in negative ion mode. Thus, the analytical identification process primarily focused on this mode, with positive ion mode serving as a supplementary confirmation. A total of 64 major chromatographic peaks were detected in the 50% methanol extract of Swertia davidi Franch, with 55 compound structures identified. The identified compounds were predominantly composed of iridoids and xanthones, with a few saccharides, nucleosides, triterpenoids, and fatty acids, among other compound types. Furthermore, several complex iridoid structures were discovered, including Senburiside III, Senburiside II, Swertianin, and acylated Swertianin (Table S1).

### 3.2. Network Pharmacology Combined with Bioinformatics Analysis Reveals That the Key Component Swertianin from Swertia Davidi Franch May Regulate PPARG Expression

**Figure 3:**
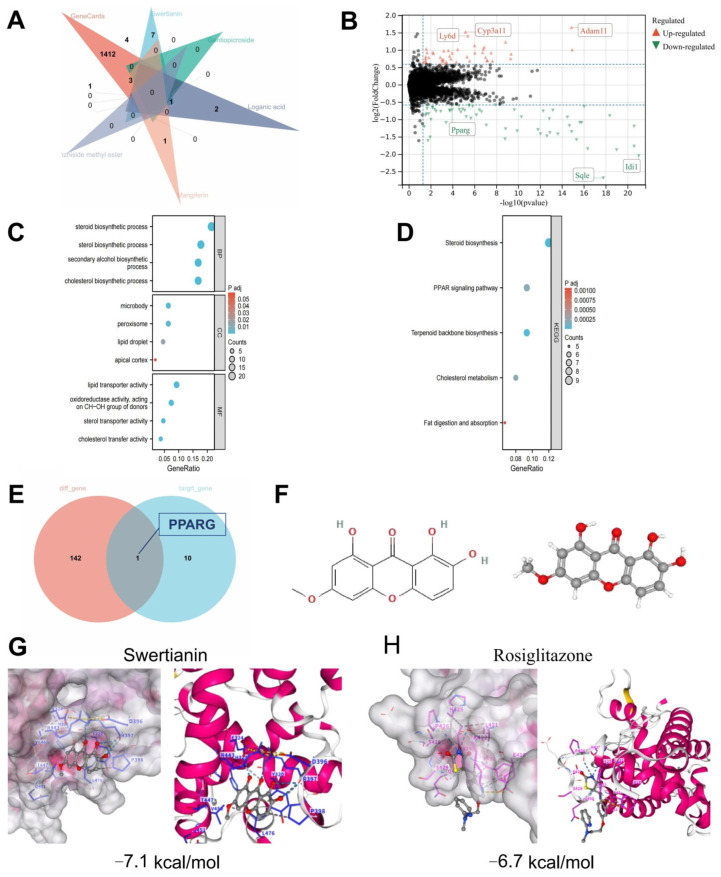
Bioinformatics and molecular simulation analysis of the interaction between components of Swertia davidi Franch and key targets of MASLD. Note: (**A**) The intersection between representative components of Swertia davidi Franch in the TCSMP database and target genes related to MASLD-associated diseases; (**B**) Differential analysis between normal liver tissue samples (*n* = 60) and MASLD liver tissue samples (*n* = 60) in the GEO MASLD-related dataset GSE149863; (**C**,**D**) Bubble plots of GO and KEGG enrichment analysis for DEGs; (**E**) Intersection analysis of DEGs and target genes for the treatment of MASLD with the aforementioned compounds including Swertianin; (**F**) 2D and 3D structures of Swertianin from the PubChem database; (**G**,**H**) Results of molecular docking experiments demonstrating the molecular docking results using AutoDock software, especially the binding affinity of Swertianin and Rosiglitazone to PPARG and the docking results with other core targets.

Previous studies have demonstrated that Swertianin possesses a unique xanthone structure, including a catechol moiety and a fully conjugated system, making it a promising antioxidant agent [42]. In order to explore the potential of Swertia davidi Franch in treating MASLD, non-representative saccharides, nucleosides, triterpenoids, and fatty acids obtained from UPLC-Q/TOF-MS analysis were excluded. The remaining compounds were subjected to structural retrieval on the PubChem website, resulting in the identification of 11 known 2D structures representing compounds from Swertia davidi Franch. These compounds include Swertianin, amarogentin, gentiopicroside, loganic acid, mangiferin, amaroswerin, shanzhiside methyl ester, Sweroside, swertiamarin, Senburiside III, and Senburiside IV. Subsequently, the 11 compounds were entered into the TCMSP database to retrieve their potential targets, using thresholds of oral bioavailability (OB) ≥ 30% and drug-likeness (DL) score ≥ 0.18. The results showed that among the 11 compounds, only Swertianin, gentiopicroside, loganic acid, mangiferin, and shanzhiside methyl ester had known target information [43,44,45]. Disease-related target points for MASLD were obtained from the GeneCards website. By intersecting the target points of the aforementioned five compounds with disease-related target points of MASLD, it was found that Swertianin, loganic acid, mangiferin, and shanzhiside methyl ester shared intersectional target points with the disease, with Swertianin showing a prominent overlap of targets, including PPARG, NOS2, GSK3B, MAOA, PTGS2, ESR1, and DPP4 (Figure 3A). This analysis revealed that the main active components, such as Swertianin, may interact with multiple biological targets, including key inflammation regulatory factors such as PPARG. These results provide a crucial molecular basis for further investigating the therapeutic mechanisms of Swertia davidi Franch in MASLD.

Subsequently, to screen for the key targets of Swertia davidi Franch in treating MASLD further, we downloaded a transcriptional dataset related to MASLD from the GEO database. Through the analysis of DEGs, a total of 143 DEGs significantly associated with MASLD were identified. These DEGs showed significant expression differences between MASLD liver tissue samples and normal liver tissue samples, including 69 upregulated genes and 74 downregulated genes (Figure 3B). The discovery of these DEGs provides a novel perspective for understanding the molecular mechanisms of MASLD. Through GO and KEGG pathway enrichment analysis, we found that these DEGs are mainly involved in processes such as steroid biosynthesis, lipid droplets, and the PPAR signaling pathway (Figure 3C,D).

In the final stage of our study, we intersected the DEGs with the target genes of the four aforementioned compounds, including Swertianin, used in the treatment of MASLD. Our results revealed a significant downregulation of the target gene PPARG by Swertianin in MASLD liver tissue (Figure 3B,E). Additionally, we obtained the 2D and 3D structures of Swertianin from the PubChem database and conducted molecular docking with the protein encoded by PPARG, demonstrating that Swertianin can bind to the active pocket of the PPARG protein (Figure 3F,G). The binding energy of the PPARG agonist Rosiglitazone with PPARG was −6.7 kcal/mol, while that of Swertianin was −7.1 kcal/mol. Therefore, the binding affinity of Swertianin is comparable to that of Rosiglitazone, suggesting its potential to bind to PPARG. Several studies have indicated that in rodent models and MASLD patients, the hepatic expression of the PPARG is negatively correlated with liver fat content and disease severity, highlighting the crucial role of PPARG in the occurrence and progression of MASLD [46,47,48].

Through this comprehensive series of analyses, our study provides a significant molecular basis for the therapeutic mechanism of Swertianin from Swertia davidi Franch in the treatment of MASLD.

### 3.3. Swertianin Activates PPARG Expression to Inhibit M1-Type Macrophage Polarization

**Figure 4:**
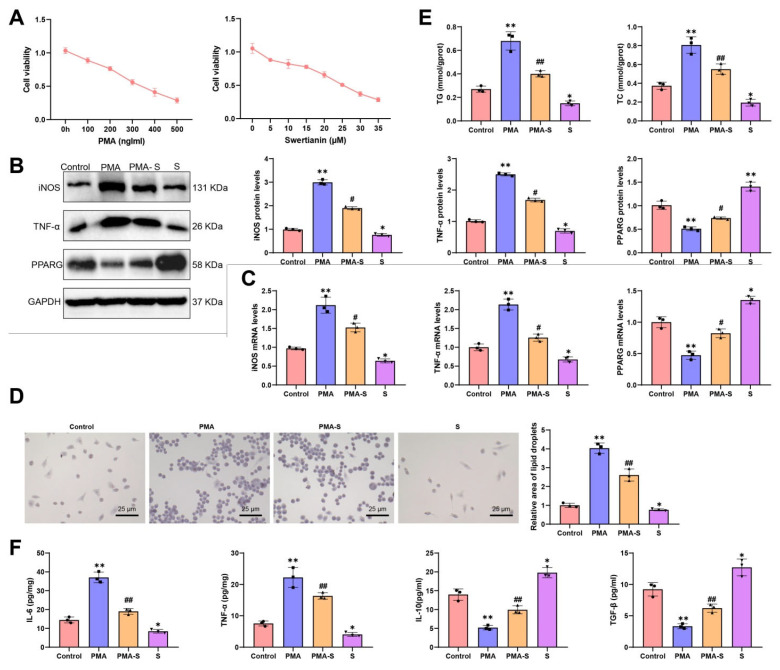
Analysis of the effects of Swertianin on THP-1-derived macrophages. Note: (**A**) CCK-8 assay assessing cytotoxicity of various concentrations of PMA and Swertianin on THP-1 cells; (**B**) Western blot results showing the expression levels of M1 markers iNOS, TNF-α, and PPARG in macrophages after Swertianin treatment; (**C**) RT-qPCR results showing the expression levels of TNF-α and iNOS in Swertianin-treated macrophages; (**D**) Results of Oil Red O staining demonstrating the quantity and area of lipid droplets in macrophages treated with Swertianin, with a scale bar of 25 μm; (**E**) Biochemical analysis showing intracellular levels of TG and TC in THP-1 cells after treatment; (**F**) ELISA results showing the levels of TNF-α, IL-6, IL-10, and TGF-β inflammatory factors in the culture supernatant of macrophages treated with Swertianin. Data are presented as mean ± standard deviation, with cell experiments repeated three times. * *p*  <  0.05, ** *p*  <  0.01 vs. Control group; # *p*  <  0.05, ## *p*  <  0.01 vs. PMA group.

Our study delves into the effects of Swertianin on macrophages. Initially, we cultured THP-1 cells in the laboratory and induced their differentiation into macrophages by treating them with 100 ng/mL phorbol-12-myristate-13-acetate (PMA, MedChemExpress, Monmouth Junction, NJ, USA) for 48 h. Subsequently, we treated the macrophages with Swertianin at a concentration of 10 μM. Based on the results of the CCK-8 cytotoxicity assay, treatment with 100 ng/mL PMA and 10 μM Swertianin exhibited no significant cytotoxic effects on THP-1 cells (Figure 4A). Subsequently, in the Western blot and RT-qPCR experiments, we assessed the expression of M1 macrophage markers (such as iNOS and TNF-α) and PPARG in Swertianin-treated macrophages. Our findings indicated that post-PMA treatment, the mRNA and protein levels of iNOS and TNF-α significantly increased, while the expression of PPARG decreased notably. In comparison to the PMA group, post-Swertianin treatment, there was a significant decrease in the mRNA and protein levels of iNOS and TNF-α, coupled with a significant increase in PPARG expression. Furthermore, treatment with Swertianin alone also significantly decreased the expression of iNOS and TNF-α while increasing PPARG expression compared with the control group (Figure 4B,C).

Additionally, we conducted Oil Red O staining experiments to evaluate the impact of Swertianin on lipid accumulation in macrophages. The results revealed that post-PMA induction, there was a significant increase in the number and area of lipid droplets in macrophages. Notably, Swertianin alone also significantly decreased lipid droplet accumulation compared with the control (Figure 4D). The intracellular levels of TG and TC in macrophages were also measured. The results showed that the TG and TC levels were significantly elevated in the PMA group compared with controls, whereas Swertianin significantly reduced the TG and TC levels in the PMA-S group (Figure 4E). In ELISA experiments, we observed a significant reduction in the levels of inflammatory factors (such as TNF-α and IL-6) in the culture supernatant of macrophages treated with Swertianin, with TNF-α decreasing by approximately 50% and IL-6 by approximately 60% (Figure 4F). Moreover, Swertianin alone also significantly reduced TNF-α and IL-6 levels compared with the control (Figure 4F). We further examined the expression of M2 macrophage markers. The results showed that IL-10 and TGF-β levels in the culture supernatant were significantly elevated following Swertianin treatment (Figure 4F). Collectively, these results demonstrate that Swertianin effectively suppresses M1 macrophage polarization and promotes an M2-like phenotype, accompanied by PPARG activation, reduced lipid accumulation, and decreased pro-inflammatory cytokine production. Additionally, we constructed PPARG-knockdown THP-1 cells (Figure S1A). Following treatment with PMA and Swertianin, iNOS and TNF-α mRNA and protein levels remained elevated in PPARG-knockdown cells compared with controls (Figure S1B–E), indicating that the regulatory effect of Swertianin on macrophage polarization is PPARG-dependent. Therefore, Swertianin may impact the functionality of macrophages by activating PPARG expression, potentially offering a new mechanism for treating MASLD and mitigating lipid deposition, thereby providing novel insights for MASLD therapeutic strategies.

### 3.4. Swertianin Activates PPARG Expression to Suppress M1-Type Macrophage Polarization and Improve MASLD Lipid Deposition

**Figure 5:**
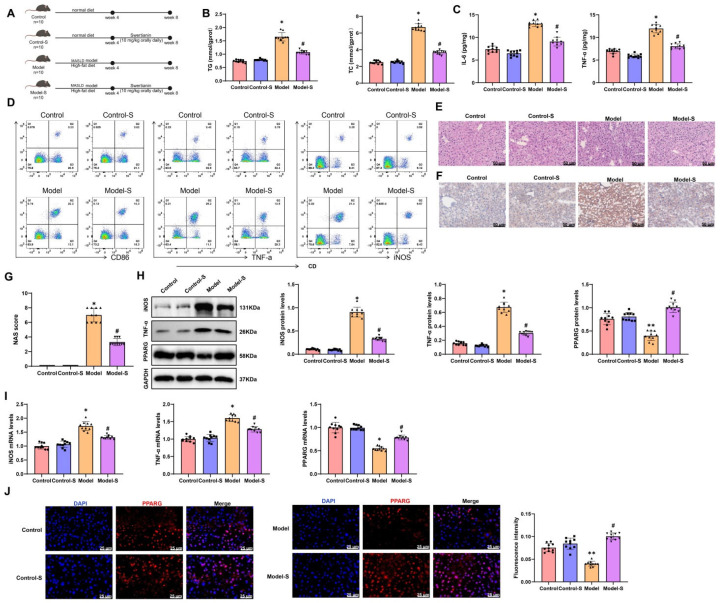
Analysis of the therapeutic effects of Swertianin on a MASLD mouse model. Note: (**A**) Experimental flowchart illustrating the overall experimental procedure of establishing a MASLD mouse model and treating it with Swertianin; (**B**) Biochemical analysis results showing changes in serum TG and TC levels in mice after treatment; (**C**) ELISA detection results showing changes in serum levels of inflammatory factors (such as TNF-α and IL-6); (**D**) Evaluation of M1 polarization marker CD86, iNOS, and TNF-αusing flow cytometry; (**E**) Pathological analysis results of liver tissue showing H&E-stained liver tissue sections, with a scale bar of 50 μm; (**F**) Oil Red O staining to assess lipid accumulation levels, with a scale bar of 50 μm; (**G**) NAS scoring of liver histopathology; (**H**,**I**) Analysis of M1 macrophage markers and PPARG expression in liver tissue demonstrating Western blot and RT-qPCR results; (**J**) PPARG expression in liver tissue. * *p*  <  0.05, ** *p*  <  0.01 vs. Control group; # *p*  <  0.05 vs. Model group. Data are presented as mean ± standard deviation, *n* = 10.

This study evaluated the therapeutic effect of Swertianin by constructing an MASLD mouse model. Forty male C57BL/6 mice were randomly divided into four groups, each containing 10 mice: (1) normal diet (Control), (2) normal diet + Swertianin (Control-S), (3) high-fat diet (Model), and (4) high-fat diet + Swertianin (Model-S). The Model and Model-S groups were successfully established as MASLD models after four weeks of feeding the patients a high-fat diet. Following treatment, a series of biochemical and molecular biology analyses were conducted on the mice (Figure 5A, experimental flowchart).

In terms of biochemical analysis, we measured the levels of TG and TC in mice serum. The results showed that compared with the Control group, the Model group had significantly increased levels of TG and TC. In contrast, the Model-S group exhibited significant reductions in TG and TC levels compared with the Model group (Figure 5B, *p* < 0.05). Furthermore, ELISA testing revealed that the IL-6 and TNFα levels in the Model group were significantly higher than those in the Control group, while the IL-6 and TNFα levels in the Model-S group were significantly lower than those in the Model group. No significant differences were observed between the Control and Control-S groups (Figure 5C). Flow cytometry analysis of the M1 macrophage markers CD86, iNOS, and TNF-α showed a significant increase in CD86, iNOS, and TNF-α levels in the Model group compared with the Control group, with the Model-S group demonstrating significantly lower CD86, iNOS, and TNF-α levels than the Model group. No significant differences were found between the Control and Control-S groups (Figure 5D). Additionally, immunofluorescence staining was performed to distinguish between monocyte-derived macrophages and resident Kupffer cells in liver tissues. Compared with the Control group, the Model group exhibited increased infiltration of monocyte-derived macrophages, while the Model-S group showed reduced infiltration (Figure S2), suggesting that Swertianin primarily affects monocyte-derived macrophages.

In the pathological analysis, we conducted H&E staining on the liver tissues of mice. The Control group exhibited no signs of hepatocellular fat degeneration or necrosis, with no lipid droplets observed within the hepatocytes. The liver sinusoids were clear and well-organized. In contrast, the Model group of mice showed severe hepatocellular fat degeneration. The hepatocytes appeared enlarged with numerous variably sized vacuolar lipid droplets within the cytoplasm, a result of nuclear compression towards the cell periphery. The compressed liver sinusoids and disorganized arrangement of liver cords were evident. In the Model-S group, there was a significant reduction in the degree of hepatocellular fat degeneration, with a decrease in vacuolation of lipid droplets in the cytoplasm; however, large lipid droplets were still visible (Figure 5E). Oil Red O staining was used to assess the impact of Swertianin on lipid deposition. The results revealed a significant increase in hepatic lipid deposition in the Model group compared with the Control group. Conversely, the Model-S group exhibited a significant reduction in hepatic lipid deposition compared with the Model group (Figure 5F). According to the Brunt scoring system devised by the Pathology Committee of the NASH Clinical Research Network (NASH-CRN), the histological score of the Model-S group was significantly lower than that of the Model group, indicating that Swertianin alleviates hepatic injury and holds potential clinical value (Figure 5G). Further analysis through Western blot and RT-qPCR assessed the expression of M1-type macrophage markers in liver tissues. The findings demonstrated a significant increase in iNOS and TNF-α expression in the liver tissues of Model mice compared with the Control group, while the Model-S group displayed significantly lower expression levels of iNOS and TNF-α compared with the Model group (Figure 5H,I). Moreover, compared with the Control group, a significant decrease in PPARG expression was observed in the liver tissues of Model mice, whereas the Model-S group exhibited significantly higher PPARG expression than the Model group (Figure 5H,I). Immunofluorescence staining of PPARG in mouse liver tissues revealed a significant reduction in PPARG expression in the liver tissues of Model mice compared with the Control group, with the Model-S group showing significantly increased PPARG expression compared with the Model group (Figure 5J).

These results suggest that Swertianin may activate PPARG expression, effectively improving hepatic lipid deposition and inflammatory conditions in the MASLD mouse model and thereby alleviating liver damage. This provides new insights into potential mechanisms and drug candidates for treating MASLD.

## 4. Discussion

In the pathogenesis of MASLD, M1 macrophages play a crucial role in inflammation and liver damage by releasing pro-inflammatory factors [49,50,51]. This study demonstrates the significant anti-inflammatory effect of Swertianin, which effectively inhibits the polarization of M1 macrophages. Certain plant extracts can reduce inflammatory responses by modulating macrophage activity. A unique aspect of this study is the elucidation that Swertianin achieves this effect by activating the PPARG pathway, providing new insights into the role of PPARG in regulating macrophage polarization. Previous research has mainly focused on the role of PPARG in adipocyte differentiation and insulin sensitivity, while this study expands its potential application in immune modulation.

In this study, treatment with Swertianin significantly improved the pathological status of MASLD in mice, reducing lipid deposition in the liver. This outcome is consistent with existing research where various natural compounds have been reported to improve hepatic lipid metabolism. However, this study further reveals the specific mechanism of action of Swertianin, namely reducing the polarization of M1 macrophages and the release of inflammatory mediators through the activation of PPARG. This clarification of the mechanism not only aids in understanding the pathway of Swertianin’s action but also underscores the potential of targeting PPARG in the treatment of MASLD.

In this study, treatment with Swertianin significantly reduced the expression of inflammatory factors, directly linked to its ability to inhibit M1-type macrophage polarization. This finding aligns with other research, suggesting that natural extracts can serve as anti-inflammatory agents. However, the unique aspect of Swertianin’s effectiveness lies in its specific regulation of these inflammatory factors through the PPARG pathway, a feature not commonly observed in previous studies. Through comparison, the distinct anti-inflammatory mechanism of Swertianin offers a new perspective for considering therapeutic strategies for chronic inflammatory diseases.

PPARG, as a nuclear receptor, has been extensively studied in regulating lipid and glucose metabolism [52,53,54]. In this study, Swertianin inhibits the polarization of M1 macrophages by activating PPARG, presenting a novel application perspective of PPARG in immune modulation. This complements strategies in other studies where PPARG agonists have been used to treat metabolic syndrome, but this study further expands the potential value of PPARG agonists in anti-inflammatory and immune modulation aspects. From the GeneCards database, we identified disease-related targets associated with MASLD, among which PPARG and iNOS appear to be key regulatory nodes. In MASLD, activation of PPARG promotes fatty acid oxidation and storage, thereby reducing hepatic lipid accumulation [55]. NOS2, on the other hand, is primarily involved in the inflammatory response in MASLD. The enzyme it encodes, iNOS, catalyzes the production of nitric oxide (NO), which plays a role in regulating vascular tone and immune responses [56]. However, activation of NOS2 may contribute to exacerbation of inflammation, and its direct role in the treatment of NAFLD is less clearly defined compared with PPARG. Therefore, although NOS2 also plays an important role in NAFLD, the therapeutic mechanism of PPARG is currently better understood and supported by more robust clinical evidence.

Although Swertianin exhibits promising bioactivity in this study, further research is needed to investigate its pharmacokinetic properties and bioavailability in vivo. The safety and efficacy of Swertianin compared with other known PPARG agonists may be crucial factors in its future clinical application [52,57,58]. Additionally, understanding its metabolic pathways in the human body and potential drug interactions are essential areas for future research.

Given the significant therapeutic effects of Swertianin in animal models, its transition to the clinical trial phase is feasible to a certain extent. However, the implementation of clinical trials necessitates careful considerations in dose determination, patient selection criteria, and evaluation of its long-term safety and efficacy. Future studies should concentrate on these aspects to ensure the successful conduct of clinical trials and the generation of scientific outcomes.

The present study successfully elucidated the therapeutic mechanism of Swertianin, the main component of Swertia davidi Franch, on MASLD through UPLC-Q/TOF-MS and network pharmacology analysis (Graphic abstract). Experimental results demonstrated that Swertianin effectively inhibits the polarization and activation of M1-type macrophages, reduces the production of inflammatory factors, and significantly decreases lipid accumulation. These effects may be mediated through the activation of key targets such as PPARG, thereby ameliorating the pathological condition of MASLD. In animal models, treatment with Swertianin markedly improved hepatic lipid deposition and inflammatory response, consequently reducing liver damage.

By investigating the impact of Swertianin in activating the PPARG pathway on the inhibition of M1-type macrophage polarization, this study introduces a novel therapeutic strategy for MASLD. Utilization of Swertianin alleviated liver inflammation and fat deposition in the mouse model, demonstrating significant therapeutic efficacy. From a scientific perspective, this discovery broadens our comprehension of the role of PPARG in immune regulation and underscores the potential value of plant extracts in modulating inflammation associated with chronic diseases. Clinically, if these findings can be validated in humans, Swertianin or its derivatives could emerge as effective therapeutic agents for MASLD, particularly offering a new option in cases where traditional treatments are ineffective or insufficient.

While this study yielded positive results in animal models, there are limitations that need to be acknowledged. Firstly, although mouse models can simulate certain aspects of human diseases, there are physiological and metabolic differences between mice and humans, which may limit the applicability of the results to humans. Additionally, the use of the THP-1 monocytic cell line rather than primary macrophages to evaluate the effect of Swertianin may not fully replicate the in vivo behavior of native macrophages. Notably, IFN-γ is often used in combination with PMA to induce M1 polarization, as it binds to IFN-γ receptors on macrophages, activates the JAK-STAT signaling pathway, and upregulates pro-inflammatory genes, thereby promoting M1 polarization [59]. Future studies combining IFN-γ, PMA, and Swertianin will provide more clinically relevant insights. Secondly, the purity and dosage of Swertianin used in the study may be challenging to replicate in clinical applications accurately, and long-term safety assessments have not been conducted. Additionally, the study did not investigate the potential side effects of Swertianin or its interactions with other medications, which are crucial considerations for its eventual clinical application. Lastly, the cellular source of iNOS in liver tissue warrants further clarification. iNOS can be induced in hepatocytes in response to inflammatory cytokines (e.g., TNF-α, IL-1β, IFN-γ) and bacterial LPS [60]. Moreover, interactions between hepatocytes and Kupffer cells are bidirectional, with Kupffer cell-derived TNF-α inducing hepatocytic iNOS expression and hepatocyte-derived NO potentially feeding back to regulate Kupffer cell function [61]. Therefore, the observed hepatic iNOS expression may originate from multiple cell types. Future studies will aim to accurately define the cellular source of iNOS in the liver under different pathological conditions. In the gene enrichment analysis of transcriptome sequencing, we selected |log_2_FC| > 0.5 as the screening threshold to avoid missing genes with moderate expression changes that may have important biological significance. However, this relatively lenient threshold may introduce potential false positives. Therefore, in future studies, we will consider applying a more stringent cutoff (e.g., |log_2_FC| > 1) to obtain more robust results.

Future research should aim to deepen and broaden the understanding of Swertianin on various fronts. Firstly, clinical trials involving a wider range of individuals are needed to validate the effectiveness and safety of Swertianin, particularly in patients with different stages of MASLD. Exploring the potential synergistic effects of combining Swertianin with existing treatment methods is also an important direction for future research. Furthermore, studies should delve into the detailed mechanisms of Swertianin in metabolism and immune regulation to better comprehend its mode of action and optimize its therapeutic effects. This study primarily focused on a qualitative investigation of the biological effects and therapeutic potential of Swertianin. In future quantitative studies, we plan to incorporate internal standard-based normalization to enable more accurate quantification of Swertianin. Through these comprehensive investigations, it is hoped that more effective and safer treatment options can be developed for MASLD and other related chronic inflammatory diseases.

## Figures and Tables

**Table 1:** Primer sequences for cell qPCR.

Genes	Forward Sequence (5′-3′)	Reverse Sequence (5′-3′)
GAPDH	GGAGCGAGATCCCTCCAAAAT	GGCTGTTGTCATACTTCTCATGG
TNF-α	CCTCTCTCTAATCAGCCCTCTG	GAGGACCTGGGAGTAGATGAG
iNOS	AGGGACAAGCCTACCCCTC	CTCATCTCCCGTCAGTTGGT
PPARG	GGGATCAGCTCCGTGGATCT	TGCACTTTGGTACTCTTGAAGTT

**Table 2:** Primer sequences for liver tissue qPCR.

Genes	Forward Sequence (5′-3′)	Reverse Sequence (5′-3′)
GAPDH	AGGTCGGTGTGAACGGATTTG	GGGGTCGTTGATGGCAACA
TNF-α	CTGAACTTCGGGGTGATCGG	GGCTTGTCACTCGAATTTTGAGA
iNOS	ACATCGACCCGTCCACAGTAT	CAGAGGGGTAGGCTTGTCTC
PPARG	CTCCAAGAATACCAAAGTGCGA	GCCTGATGCTTTATCCCCACA

## Data Availability

All data can be provided as needed.

## Supplementary Materials

The following supporting information can be downloaded at: https://www.mdpi.com/article/10.3390/genes16060693/s1, Figure S1: Swertianin’s regulation of macrophage polarization is PPARG-dependent; Figure S2: Therapeutic efficacy of Swertianin in MASLD mouse models; Table S1: Identification results of major chromatographic peaks in total ion chromatogram under negative ion mode (ordered by retention time).
