# Supplementary material for: Swertianin Suppresses M1 Macrophage Polarization and Inflammation in Metabolic Dysfunction-Associated Fatty Liver Disease via PPARG Activation
Source: Genes (Basel). 2025 Jun 6;16(6):693. doi: 10.3390/genes16060693 (PMC12193489; doi:10.3390/genes16060693)
Supplement: Supplementary file 1 [file genes-16-00693-s001.zip › genes-3517733-supplementary.pdf]

Table S1. Identification results of major chromatographic peaks in total ion chromatogram under negative ion mode (ordered by retention time).

Number	Component name	Chemical formula	Observation retention time (min)	Molecular mass number (Da)	Observed m/z	Mass error (ppm)	Adduct form	Main fragment ions	Structural formula (example)
1	Sucrose	C12H22O11	0.72	342.1162	341.1063<br>387.1115	-2.1	【M-H】$^-$;<br>【M+HCOO】$^-$	179.0544	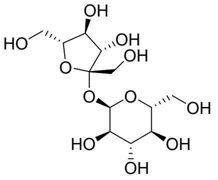
2	β-Methyl-Garcinia-lacton	C7H8O7	0.95	204.0270	203.0186	-3.0	【M-H】-	--	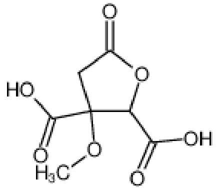
3	Quinic acid	$C_6H_8O_7$	1.00	192.0270	191.0191	-0.5	【M-H】-	155.9550	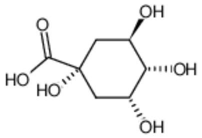

4	Uridine	C9H12N2O6	1.36	244.0695	243.0606	-4.5	【M-H】$^-$	111.0190-	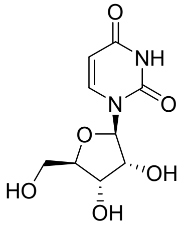
5	Thymidylic acid	C10H12N2O6	1.78	256.0695	255.0610	-1.9	【M-H】$^-$	211.0716	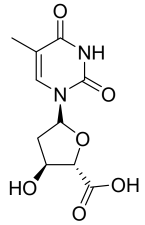
6	Thymidine deoxyribonucleoside isomer	C10H12N2O6	1.88	256.0695	255.0610	-1.9	【M-H】$^-$	211.0716	Structure omitted
7	Gaultherioside	C13H24O10	3.73		339.1304	3.8	【M-H】$^-$	295.1034<br>207.0889	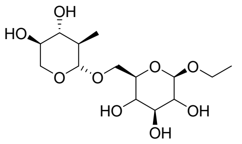
8	Gentianolic acid glucoside	C13H16O9	4.23	316.0794	315.0716	-1.9	【M-H】$^-$	153.0204	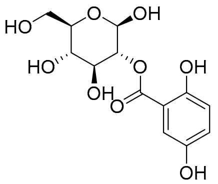
9	Droxidopa	C9H11NO5	4.40	213.0637	212.0576	8.0	【M-H】$^-$	168.0664	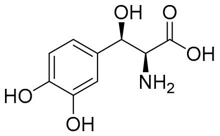

10	Shanzhiside methyl ester	C17H26O11	4.71	406.1475	405.1379	-4.4	【M-H】-	179.0588	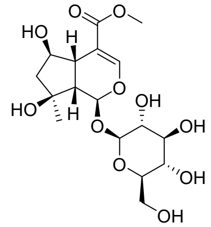
11	Loganic acid glucoside	C22H34O15	4.86	537.1819	537.1804	-0.5	【M-H】-	375.1283；213.0810；169.0945	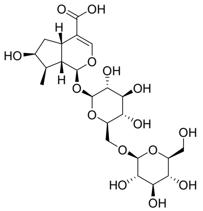
12	Loganic acid	C16H24O10	4.93	375.1291	375.1283	-1.0	【M-H】-	213.0810；169.0945	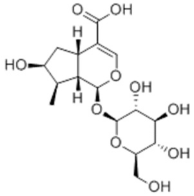
13	Gallic acid	C7H6O5	5.19	170.0215	169.0147	1.0	【M-H】-	125.0245	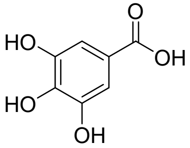
14	Cyclosporin	C16H22O11	5.38	389.1084	389.1085	0.1	【M-H】-	345.1213；183.0667	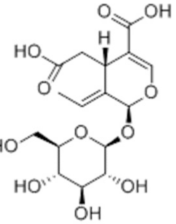

15	Glucosylgentiopicroside	C22H30O14	5.54	518.1636	563.1595	-3.0	【M-H】-	341.109<br>5<br>221.068<br>0<br>179.056<br>0	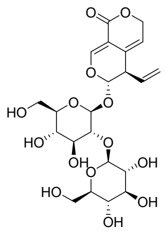
16	Swertiamarin	C16H22O10	5.65	373.1135	373.1158<br>;<br>419.1183	2.3	【M-H】-	179.0595;<br>161.054<br>3;	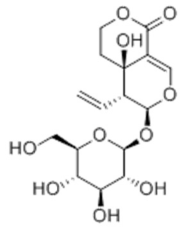
17	Mangiferin	C19H18O11	5.84	422.0849	421.0789	4.3	【M-H】-	403.0672<br>331.0446<br>301.0367<br>271.0250<br>259.0239	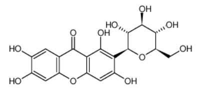
18	Isomangiferin	C19H18O11	6.15	422.0849	421.0789	4.3	【M-H】-	403.0672<br>331.0446<br>301.0367<br>271.0250<br>259.0239	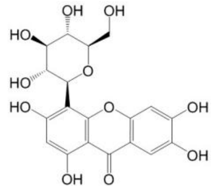

19	Gentiopicroside	$C16H20O9$	6.19	356.1107	401.1084	0.0	【M-H】$^-$	355.1052<br>193.0536	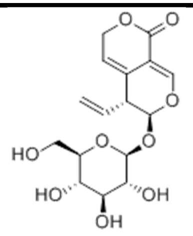
20	Sweroside	$C_{16}H_{22}O_9$	6.41	358.1264	357.1186<br>403.1248	-1.7	【M-H】$^-$	195.0679	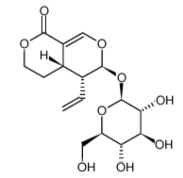
21	Norswertianolin	$C19H18O11$	7.00	422.0849	421.0789	4.3	【M-H】$^-$	259.0239	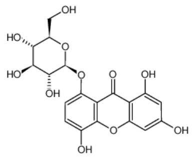
22	Orientin<br>(Isoorientin)	$C21H20O11$	7.09	448.1010	447.0947	3.8	【M-H】$^-$	357.0600<br>327.0512<br>297.0414<br>285.0413	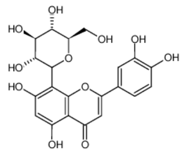
23	Isovitexin	$C21H20O10$	8.28	432.1060	431.0973	-1.2	【M-H】$^-$	341.0671<br>311.0562<br>283.0602	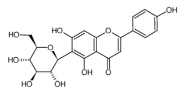
24	Swertisin	$C_{22}H_{22}O_{10}$	8.77	446.1231	445.1134	-0.2	【M-H】$^-$	325.0691<br>297.0379	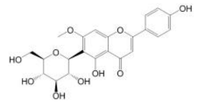
25	Unknown	$C15H16O7$	8.89	308.0896	307.0816	-0.7	【M-H】$^-$	235.0607	

26	Unknown	C12H17NO5	9.37	255.1107	254.1036	3.1	【M-H^{-}	191.0700<br>210.1147	
27	Unknown	C35H40O18	9.44	748.2217	747.2144	1.1	【M-H】$^{-}$	597.1465<br>533.2164<br>407.0982	
28	Unknown	C12H17NO5	9.56	255.1107	254.1036	3.1	【M-H】$^{-}$	273.0415<br>210.1147	
29	Primeverosylbellidifolin	C25H28O15	10.00	568.1430	567.1370	3.5	【M-H】$^{-}$	435.0863<br>273.0415<br>258.0163<br>230.0173	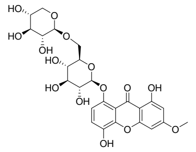
30	Primeverosylswertianin	C25H28O15	10.22	568.1430	567.1370	3.5	【M-H】$^{-}$	435.0863<br>273.0415<br>258.0163<br>230.0173	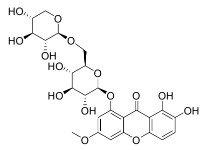
31	Metahydroxybenzoic acid	C23H28O12	10.27	496.1580	495.1515	2.4	【M-H】$^{-}$	333.0971<br>289.1078<br>271.0958<br>195.0650	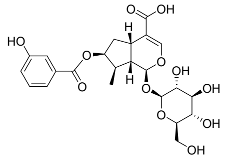

32	Gentiacaulein glucoside	C20H20O11	10.35	435.0927	435.0922	-0.5	【M-H】⁻	272.0340;<br>257.0107;	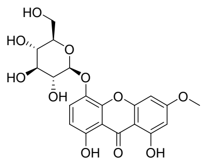
33	1,3,8-trihydroxy-2,5-dimethoxy flavone hesperidin glucoside	C26H30O16	10.65	598.1534	597.1465	1.5	【M-H】⁻	567.1370；<br>288.0259；<br>273.0415;<br>258.0163;<br>230.0173;	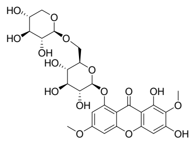
34	Amaroswerin	C29H30O14	11.00	602.1636	601.1567	1.7	【M-H】⁻	245.0453<br>227.0361<br>201.0538	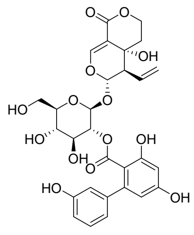
35	Unknown		11.07		836.5863		【M-H】⁻		
36	Amarogentin	C29H30O13	11.43	586.1690	585.1618	1.7	【M-H】⁻	245.0453<br>227.0361<br>201.0538	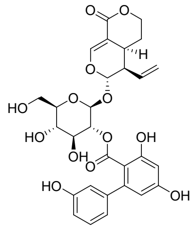
37	1,3,6-trihydroxy-4,7-dimethoxy flavone	C15H12O7	11.51	304.0583	303.0505	0.0	【M-H】⁻	288.0259；<br>287.0571；	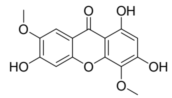

								272.0340；<br>257.0107；	
38	2-O-(2'-hydroxyphenyl-4',6'-dihydroxybenzoyl)-gentiobiosi de	C29H28O13	11.55	584.1530	583.1424	1.7	【M-H】-	407.0941<br>245.0453	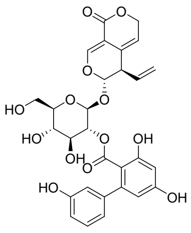
39	12,8-dihydroxy-1,6-dimethoxy flavone	C15H12O6	11.93	288.0634	287.0571	0.7	【M-H】-	272.0306<br>257.0074<br>229.0152	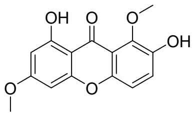
40	Senburiside IV	C36H42O19	12.21	778.2321	777.2256	1.8	【M-H】-	615.1710<br>477.1407<br>451.0653<br>409.1295<br>257.0468<br>213.0548	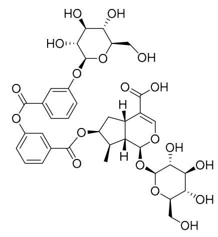
41	Trihydroxyxanthone	C13H8O5	13.60	244.0372	243.0298	2.1	【M-H】-	199.0407	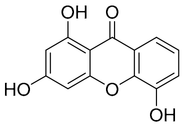
42	Desmethylbellidifolin	C13H8O6	14.57	260.0321	259.0239	-1.5	【M-H】-	231.0247<br>215.0352	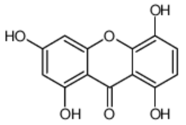

43	Swertianin<br>2-O-α-L-rhamnopyranosyl-(1→2)-β-D-xylopyranoside	C25H28O14	14.17	552.1479	551.1428	4.9	【M-H】-	405.0802<br>387.0712<br>273.0415<br>257.0170	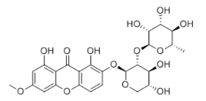
44	Senburiside III	C47H52O23	14.30	984.2899	983.2804	-1.7	【M-H】-	777.2256；<br>615.1710；<br>477.1407；<br>453.0813；<br>409.1295；<br>257.0468；<br>213.0548;	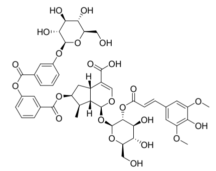
45	Senburiside IV	C46H50O22	14.50	954.2794	953.2715	-1.7	【M-H】-	777.2256；<br>615.1710；<br>477.1407；<br>453.0813；<br>409.1295；<br>257.0468；<br>213.0548;	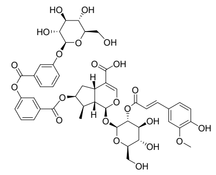
46	Senburiside II	C30H32O14	14.40	616.1792	615.1710	-0.7	【M-H】-	477.1407；<br>453.0813；<br>409.1295；	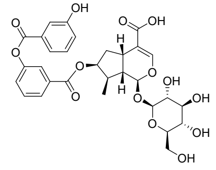

								257.0468；<br>213.0548；	
47	Norswertianin	C13H8O6	14.57	260.0321	259.0239	-1.5	【M-H】-	215.0352	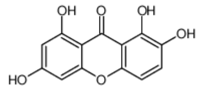
48	Hydroxyapigenin	C14H10O7	14.88	290.0427	289.0347	-0.3	【M-H】-	274.0135；<br>246.0208；	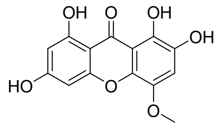
49	1,7-dihydroxy-3,8-dimethoxyflavone	C15H12O6	15.29	288.0634	287.0571	5.2	【M-H】-	272.0340；<br>257.0107；<br>229.0152；	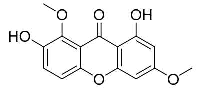
50	Bellidifolin	C14H10O6	16.74	247.0477	273.0415	5.9	【M-H】-	258.1063；<br>229.0152；	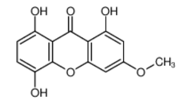
51	Swertianin	C14H10O6	17.02	247.0477	273.0415	5.9	【M-H】-	258.0163<br>230.0235<br>229.0152	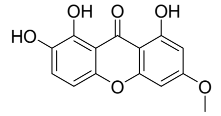
52	1,3,8-trihydroxy-2,5-dimethoxyflavone	C15H12O6	17.12	304.0583	303.0505	0.0	【M-H】-	288.0259；<br>287.0571；<br>272.0340；<br>257.0107；	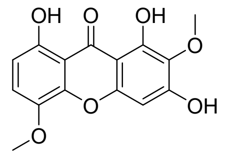

53	Isobellidifolin	C14H10O6	17.26	247.0477	273.0415	5.9	【M-H】-	258.0163；<br>230.0235；<br>229.0152；	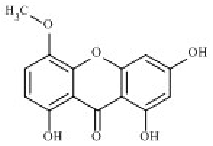
54	Hydroxyoleanolic acid (Maslinic acid)	C30H48O4	21.61		471.3475	0.1	【M-H】-	--	
55	Unknown	C39H44O15	24.29		751.2635	5.6	【M-H】-		
56	Oleanic acid	C30H48O3	25.05		455.3552<br>501.3592	5.9	【M-H】-	437.3404<br>409.3449	
57	Unknown	C39H46O15	25.61		753.2800	5.6	【M-H】-		
58	Linoleic acid	C18H32O2	26.40		279.2315	0.6	【M-H】-	--	
59	Unknown	C37H46O15	26.69		729.2775	2.3	【M-H】-		
60	Unknown	C35H56O13	26.95		683.3644	0.1	【M-H】-		
61	Palmitic acid	C18H32O2	27.35		255.2343	7.6	【M-H】-	--	
62	Oleic acid	C18H34O2	27.56		281.2473	-2.8	【M-H】-	--	
63	Ursolic acid	C30H48O3	28.14		455.3552<br>501.3592	5.9	【M-H】-	437.3404<br>409.3449	
64	Stearic acid	C18H36O2	28.72		283.2635	-0.7	【M-H】-	--	

Number	Component name	Chemical formula	Observation retention time (min)	Molecular mass number (Da)	Observed m/z	Mass error (ppm)	Adduct form	Main fragment ions	Structural formula (example)
1	sucrose	C12H22O11	0.72	342.1162	341.1063<br>387.1115	-2.1	【M-H】$^-$;<br>【M+HCOO】$^-$	179.0544	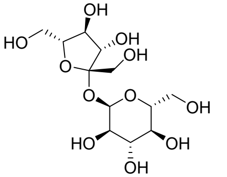
2	β-Methyl-Garcinia-lacton	C7H8O7	0.95	204.0270	203.0186	-3.0	【M-H】-	--	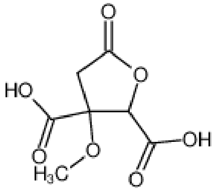
3	quinic acid	$C_6H_8O_7$	1.00	192.0270	191.0191	-0.5	【M-H】-	155.9550	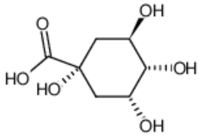

4	Uridine	C9H12N2O6	1.36	244.0695	243.0606	-4.5	【M-H】-	111.0190-	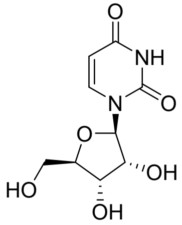
5	Thymidylic acid	C10H12N2O6	1.78	256.0695	255.0610	-1.9	【M-H】-	211.0716	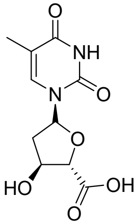
6	Thymidine deoxyribonucleoside isomer	C10H12N2O6	1.88	256.0695	255.0610	-1.9	【M-H】-	211.0716	Structure omitted
7	Gaultherioside	C13H24O10	3.73		339.1304	3.8	【M-H】-	295.1034<br>207.0889	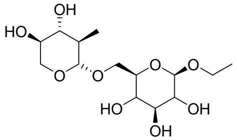
8	Gentianolic acid glucoside	C13H16O9	4.23	316.0794	315.0716	-1.9	【M-H】-	153.0204	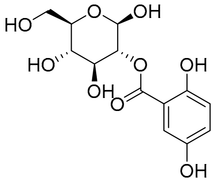
9	Droxidopa	C9H11NO5	4.40	213.0637	212.0576	8.0	【M-H】-	168.0664	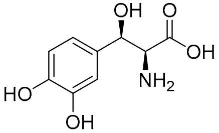

10	Shanzhiside methyl ester	C17H26O11	4.71	406.1475	405.1379	-4.4	【M-H】-	179.0588	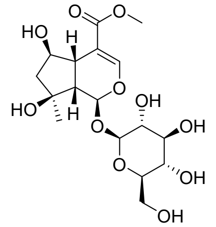
11	Loganic acid glucoside	C22H34O15	4.86	537.1819	537.1804	-0.5	【M-H】-	375.1283；213.0810；169.0945	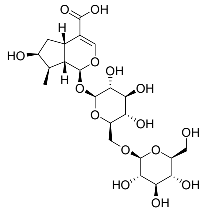
12	Loganic acid	C16H24O10	4.93	375.1291	375.1283	-1.0	【M-H】-	213.0810；169.0945	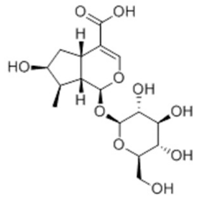
13	Gallic acid	C7H6O5	5.19	170.0215	169.0147	1.0	【M-H】-	125.0245	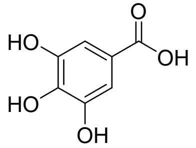
14	Cyclosporin	C16H22O11	5.38	389.1084	389.1085	0.1	【M-H】-	345.1213；183.0667	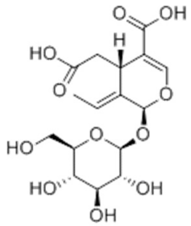

15	Glucosylgentiopicroside	C22H30O14	5.54	518.1636	563.1595	-3.0	【M-H】$^-$	341.109<br>5<br>221.068<br>0<br>179.056<br>0	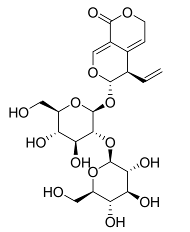
16	swertiamarin	C16H22O10	5.65	373.1135	373.1158<br>;<br>419.1183	2.3	【M-H】$^-$	179.0595;<br>161.054<br>3;	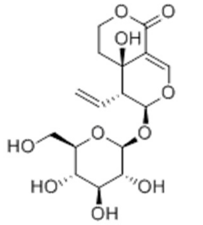
17	mangiferin	C19H18O11	5.84	422.0849	421.0789	4.3	【M-H】$^-$	403.0672<br>331.0446<br>301.0367<br>271.0250<br>259.0239	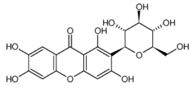
18	Isomangiferin	C19H18O11	6.15	422.0849	421.0789	4.3	【M-H】$^-$	403.0672<br>331.0446<br>301.0367<br>271.0250<br>259.0239	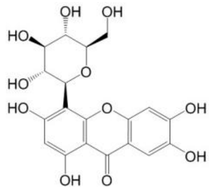

19	Gentiopicroside	$C_{16}H_{20}O_9$	6.19	356.1107	401.1084	0.0	【M-H】$^-$	355.1052<br>193.0536	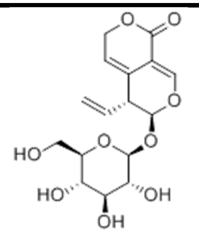
20	Sweroside	$C_{16}H_{22}O_9$	6.41	358.1264	357.1186<br>403.1248	-1.7	【M-H】$^-$	195.0679	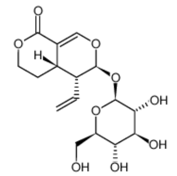
21	Norswertianolin	$C_{19}H_{18}O_{11}$	7.00	422.0849	421.0789	4.3	【M-H】$^-$	259.0239	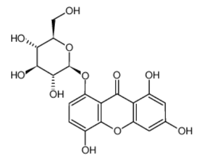
22	Orientin<br>(or Isoorientin)	$C_{21}H_{20}O_{11}$	7.09	448.1010	447.0947	3.8	【M-H】$^-$	357.0600<br>327.0512<br>297.0414<br>285.0413	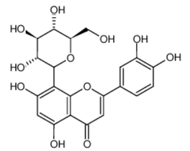
23	isovitexin	$C_{21}H_{20}O_{10}$	8.28	432.1060	431.0973	-1.2	【M-H】$^-$	341.0671<br>311.0562<br>283.0602	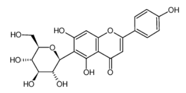
24	Swertisin	$C_{22}H_{22}O_{10}$	8.77	446.1231	445.1134	-0.2	【M-H】$^-$	325.0691<br>297.0379	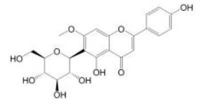
25	Unknown	$C_{15}H_{16}O_7$	8.89	308.0896	307.0816	-0.7	【M-H】$^-$	235.0607	

26	Unknown	C12H17NO5	9.37	255.1107	254.1036	3.1	【M-H$^-$	191.0700<br>210.1147	
27	Unknown	C35H40O18	9.44	748.2217	747.2144	1.1	【M-H】$^-$	597.1465<br>533.2164<br>407.0982<br>273.0415	
28	Unknown	C12H17NO5	9.56	255.1107	254.1036	3.1	【M-H】$^-$	210.1147	
29	primeverosylbellidifolin	C25H28O15	10.00	568.1430	567.1370	3.5	【M-H】$^-$	435.0863<br>273.0415<br>258.0163<br>230.0173	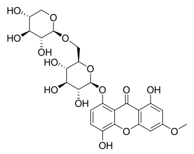
30	primeverosylswertianin	C25H28O15	10.22	568.1430	567.1370	3.5	【M-H】$^-$	435.0863<br>273.0415<br>258.0163<br>230.0173	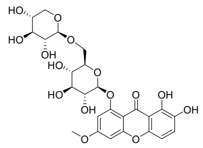
31	Metahydroxybenzoic acid	C23H28O12	10.27	496.1580	495.1515	2.4	【M-H】$^-$	333.0971<br>289.1078<br>271.0958<br>195.0650	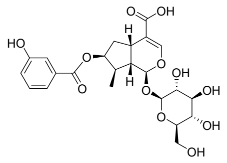

32	Gentiacaulein glucoside	C20H20O11	10.35	435.0927	435.0922	-0.5	【M-H】-	272.0340;<br>257.0107;	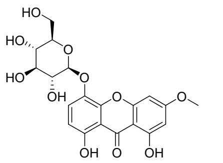
33	1,3,8-trihydroxy-2,5-dimethoxy flavone hesperidin glucoside	C26H30O16	10.65	598.1534	597.1465	1.5	【M-H】-	567.1370；<br>288.0259；<br>273.0415;<br>258.0163;<br>230.0173;	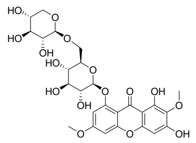
34	amaroswerin	C29H30O14	11.00	602.1636	601.1567	1.7	【M-H】-	245.0453<br>227.0361<br>201.0538	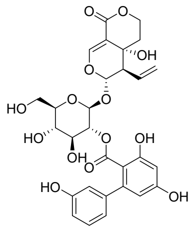
35	Unknown		11.07		836.5863		【M-H】-		
36	amarogentin	C29H30O13	11.43	586.1690	585.1618	1.7	【M-H】-	245.0453<br>227.0361<br>201.0538	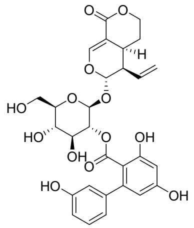
37	1,3,6-trihydroxy-4,7-dimethoxy flavone	C15H12O7	11.51	304.0583	303.0505	0.0	【M-H】-	288.0259；<br>287.0571；	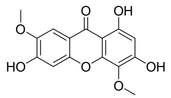

								272.0340；<br>257.0107；	
38	2-O-(2'-hydroxyphenyl-4',6'-dihydroxybenzoyl)-gentiobioside	C29H28O13	11.55	584.1530	583.1424	1.7	【M-H】-	407.0941<br>245.0453	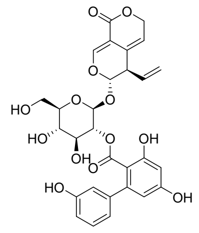
39	12,8-dihydroxy-1,6-dimethoxy flavone	C15H12O6	11.93	288.0634	287.0571	0.7	【M-H】-	272.0306<br>257.0074<br>229.0152	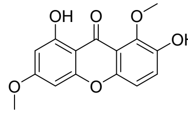
40	Senburiside IV	C36H42O19	12.21	778.2321	777.2256	1.8	【M-H】-	615.1710<br>477.1407<br>451.0653<br>409.1295<br>257.0468<br>213.0548	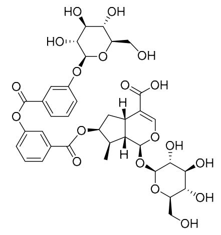
41	Trihydroxyxanthone	C13H8O5	13.60	244.0372	243.0298	2.1	【M-H】-	199.0407	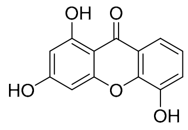
42	Desmethylbellidifolin	C13H8O6	14.57	260.0321	259.0239	-1.5	【M-H】-	231.0247<br>215.0352	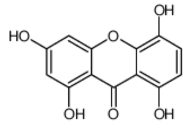

43	Swertianin 2-O-α-L-rhamnopyranosyl-(1→2)-β-D-xylopyranoside	C25H28O14	14.17	552.1479	551.1428	4.9	【M-H】-	405.0802<br>387.0712<br>273.0415<br>257.0170	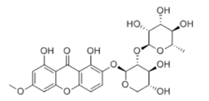
44	Senburiside III	C47H52O23	14.30	984.2899	983.2804	-1.7	【M-H】-	777.2256；<br>615.1710；<br>477.1407；<br>453.0813；<br>409.1295；<br>257.0468；<br>213.0548;	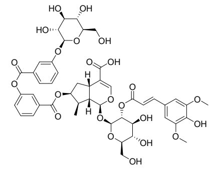
45	Senburiside IV	C46H50O22	14.50	954.2794	953.2715	-1.7	【M-H】-	777.2256；<br>615.1710；<br>477.1407；<br>453.0813；<br>409.1295；<br>257.0468；<br>213.0548;	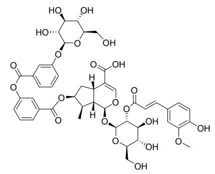
46	Senburiside II	C30H32O14	14.40	616.1792	615.1710	-0.7	【M-H】-	477.1407；<br>453.0813；<br>409.1295；	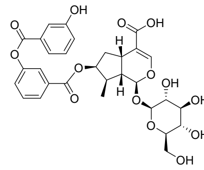

								257.0468；<br>213.0548；	
47	norswertianin	C13H8O6	14.57	260.0321	259.0239	-1.5	【M-H】$^-$	215.0352	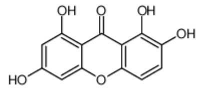
48	Hydroxyapigenin	C14H10O7	14.88	290.0427	289.0347	-0.3	【M-H】$^-$	274.0135；<br>246.0208；	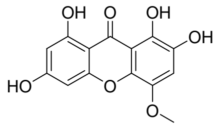
49	1,7-dihydroxy-3,8-dimethoxyflavone	C15H12O6	15.29	288.0634	287.0571	5.2	【M-H】$^-$	272.0340；<br>257.0107；<br>229.0152；	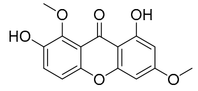
50	bellidifolin	C14H10O6	16.74	247.0477	273.0415	5.9	【M-H】$^-$	258.1063；<br>229.0152；	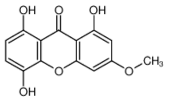
51	swertianin	C14H10O6	17.02	247.0477	273.0415	5.9	【M-H】$^-$	258.0163<br>230.0235<br>229.0152	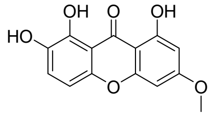
52	1,3,8-trihydroxy-2,5-dimethoxyflavone	C15H12O6	17.12	304.0583	303.0505	0.0	【M-H】$^-$	288.0259；<br>287.0571；<br>272.0340；<br>257.0107；	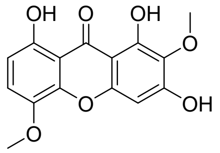

53	Isobellidifolin	C14H10O6	17.26	247.0477	273.0415	5.9	【M-H】-	258.0163；<br>230.0235；<br>229.0152；	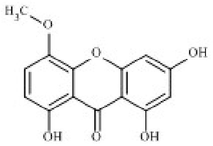
54	Hydroxyoleanolic acid (Maslinic acid)	C30H48O4	21.61		471.3475	0.1	【M-H】-	--	
55	Unknown	C39H44O15	24.29		751.2635	5.6	【M-H】-		
56	Oleanic acid	C30H48O3	25.05		455.3552<br>501.3592	5.9	【M-H】-	437.3404<br>409.3449	
57	Unknown	C39H46O15	25.61		753.2800	5.6	【M-H】-		
58	Linoleic acid	C18H32O2	26.40		279.2315	0.6	【M-H】-	--	
59	Unknown	C37H46O15	26.69		729.2775	2.3	【M-H】-		
60	Unknown	C35H56O13	26.95		683.3644	0.1	【M-H】-		
61	Palmitic acid	C18H32O2	27.35		255.2343	7.6	【M-H】-	--	
62	Oleic acid	C18H34O2	27.56		281.2473	-2.8	【M-H】-	--	
63	Ursolic acid	C30H48O3	28.14		455.3552<br>501.3592	5.9	【M-H】-	437.3404<br>409.3449	
64	Stearic acid	C18H36O2	28.72		283.2635	-0.7	【M-H】-	--	

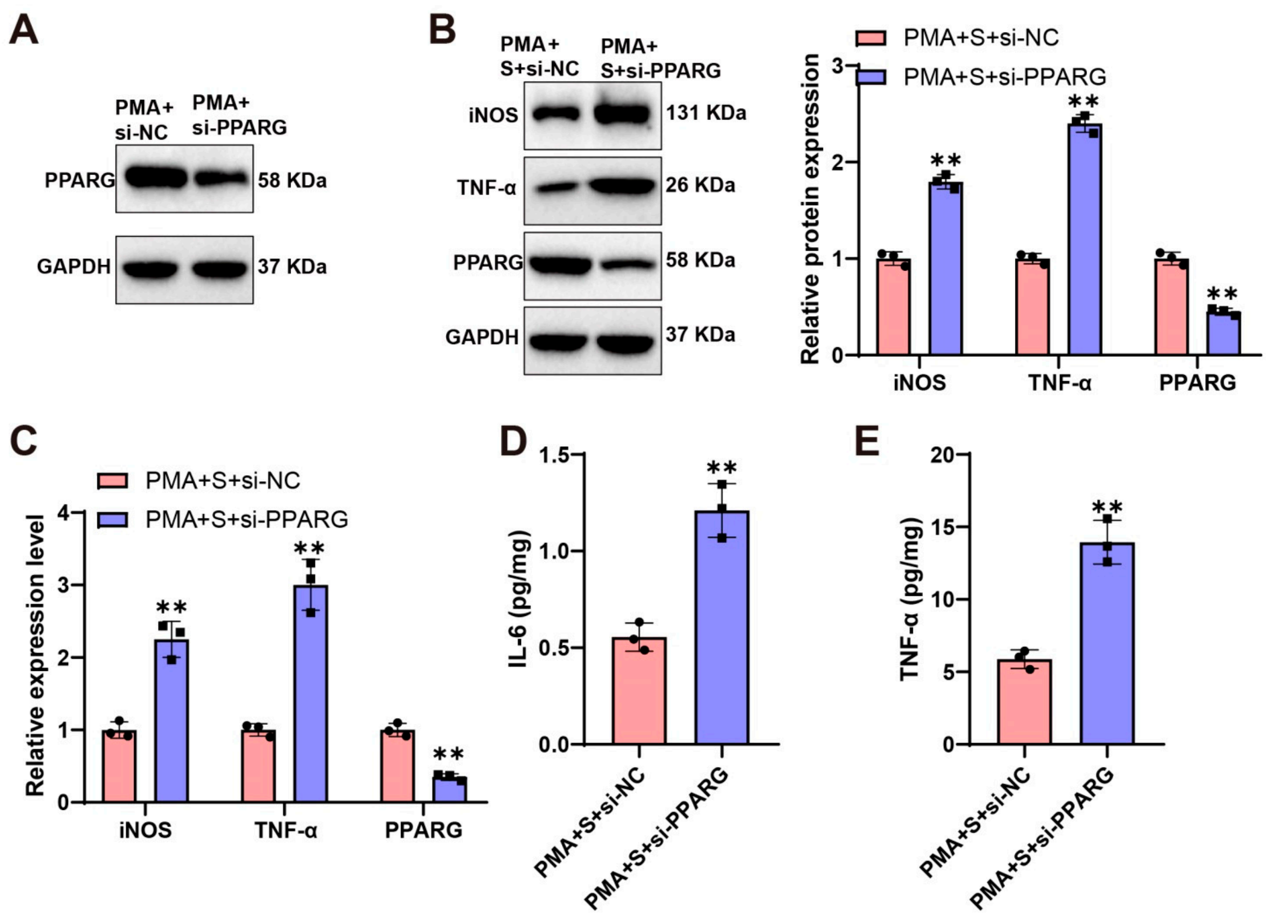

Figure S1. Swertianin's regulation of macrophage polarization is PPARG-dependent.

Note: (A) Immunoblotting of PPARG expression in sh-PPARG THP-1 cells; (B) Western Blot results showing iNOS, TNF-α, and PPARG protein levels in PPARG-knockdown macrophages treated with Swertianin; (C) RT-qPCR showing mRNA expression of iNOS and TNF-α; (D-E) ELISA detection of IL-6 and TNF-α levels in the culture supernatant of PPARG-knockdown THP-1 cells under different treatments. Only PMA+Swertianin+siRNA groups were included here as this experiment specifically aimed to assess the dependence of Swertianin's effect on PPARG.

Data are presented as mean ± SD from three independent experiments. $**p < 0.01$.

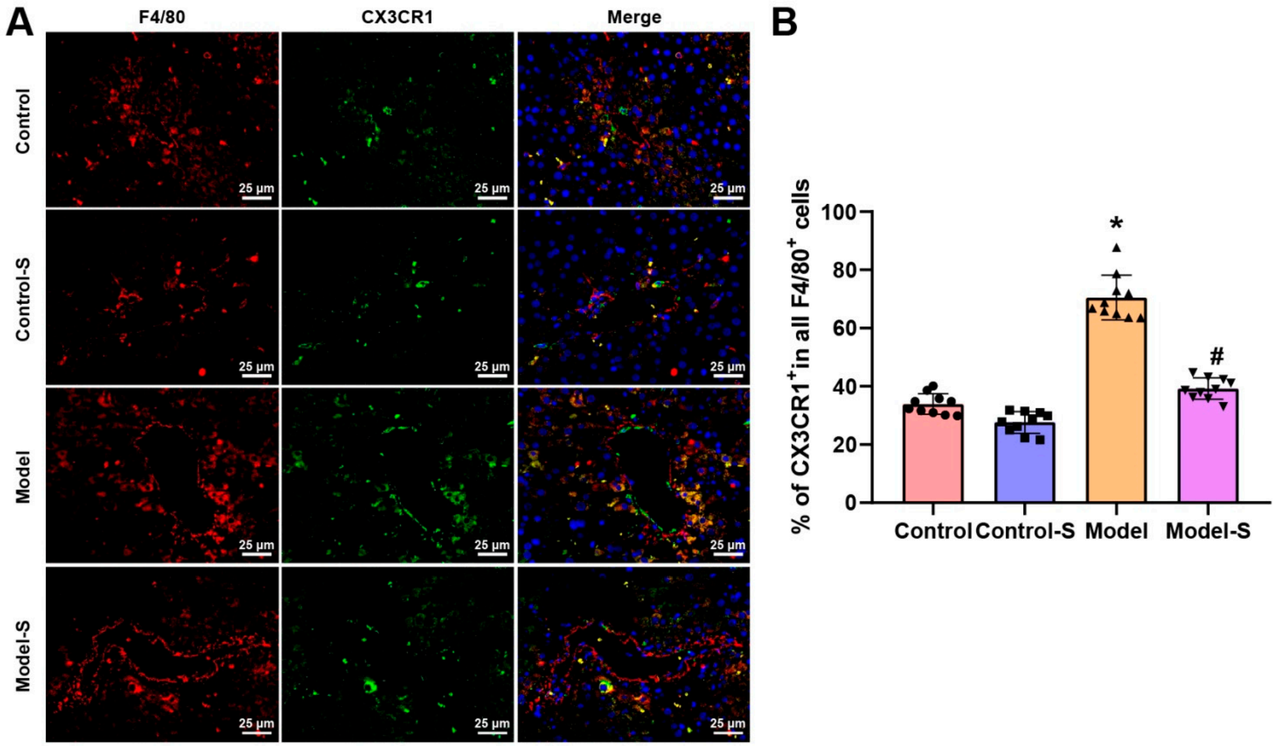

Figure S2. Therapeutic efficacy of Swertianin in MASLD mouse models.

Note: (A-B) Immunofluorescence staining showing differential distribution of Kupffer cells ($F4/80^+CX3CR1^-$) and monocyte-derived macrophages ($F4/80^+CX3CR1^+$) under various treatment conditions. *$p < 0.05$ vs. Control group; #$p < 0.05$ vs. Model group.
